# Effect of *Nigella sativa* supplementation on kidney function, glycemic control, oxidative stress, inflammation, quality of life, and depression in diabetic hemodialysis patients: study protocol for a double-blind, randomized controlled trial

**DOI:** 10.1186/s13063-021-05917-y

**Published:** 2022-02-04

**Authors:** Alireza Rahmani, Vahid Maleki, Bahram Niknafs, Omid Mohammad Tavakoli-Rouzbehani, Ali Tarighat-Esfanjani

**Affiliations:** 1 Student Research Committee, Tabriz University of Medical Sciences, Tabriz, Iran; 2 Department of Clinical Nutrition, Faculty of Nutrition and Food Science, Tabriz University of Medical Sciences, Tabriz, Iran; 3 Department of Internal Medicine, School of Medicine Imam Reza Medical Research and Training Hospital, Tabriz University of Medical Sciences, Tabriz, Iran; 4 Nutrition Research Center, Faculty of Nutrition and Food Sciences, Tabriz University of Medical Sciences, Tabriz, Iran

**Keywords:** Hemodialysis, Inflammation, *Nigella sativa*, Oxidative stress, Quality of life, Depression, Diabetes

## Abstract

**Background and objectives:**

The kidney is probably the most crucial target of microvascular damage in diabetes, which can ultimately eventuate end-stage renal disease (ESRD). Hemodialysis is the most usual way of renal replacement therapy in ESRD. Patients receiving hemodialysis are susceptible to many complications like hyperglycemia, inflammation, depression, anxiety, and poor quality of life. So, they are constrained to consume many drugs. Medicinal herbs are used in different cultures as a reliable source of natural remedies. This study aims to determine the efficacy of *Nigella sativa* (NS) oil supplementation on blood glucose, kidney function tests, inflammation, oxidative stress, quality of life, and depression in hemodialysis patients.

**Methods and analysis:**

This double-blind, randomized controlled trial will enroll 46 patients with diabetes mellitus who give hemodialysis thrice a week. Patients who have an inflammatory or infectious disease and who are receiving nonsteroidal anti-inflammatory drugs will be excluded. Patients will be randomized to the treatment and control group, which will be recommended using two soft gels of NS and paraffin oil, respectively. Laboratory tests will be assessed at baseline and end of the study, including fasting blood sugar, glycated albumin, insulin, creatinine, blood urea nitrogen, urea, uric acid, superoxide dismutase, malondialdehyde, total antioxidant capacity, high sensitive C reactive protein, and 24-h urine volume. Also, the kidney disease and quality of life and hospital anxiety and depression scale questionnaires will be evaluated.

**Discussion:**

Previous studies have reported a positive effect of *Nigella sativa* supplementation in chronic kidney disease, but there is no evidence that this plant is safe in hemodialysis patients. The results of this study can be helpful in better control of blood sugar and kidney function and reduce complications in diabetic hemodialysis patients.

**Trial registration:**

Iranian Registry of Clinical Trials . Registered on 31 May 2020

**Supplementary Information:**

The online version contains supplementary material available at 10.1186/s13063-021-05917-y.

## Introduction

Diabetes and chronic kidney disease (CKD) are pervasive diseases of the present century, increasing prevalence. Diabetes is the leading cause of renal failure, which ultimately results in the end stage of renal disease (ESRD) and the onset of dialysis or kidney transplantation [1].

By reducing infectious diseases and prolonging the population’s life, CKD has been the most acute chronic non-communicable disease for life-threatening human beings [2]. The prevalence of CKD in different countries depends on many factors, such as gender, age, and economic status. However, it is estimated that 14% of the world’s population is involved in this disease, and accordingly, its prevalence is rising [3]. The prevalence of RRT in many countries, particularly in the Asian continent, is growing, and by 2030, the number of dialysis patients reaches at least 5.4 million [4].

*Nigella sativa* (NS) is an excellent plant from Ranunculaceae and indigenous to Southwest Asia. This plant is used in Iranian traditional medicine to treat various disorders such as respiratory, gastrointestinal, hepatic, and renal disorders. Many of its therapeutic effects have been proven in previous research [5]. Pharmacological studies have shown anti-diabetic, antioxidant, and anti-inflammatory properties of NS because of this plant’s different chemical components. Among these, compounds can be pointed at thymoquinone and nigellidin [6].

About 50% of patients dependent on dialysis are diagnosed with diabetes [7] and are prescribed insulin. Glycosylation of some tissues such as glomerulus and mesangial cells is a determining factor in the progression and development of renal failure. The common indicators for evaluating glycemic status are fasting blood sugar (FBS), glycated hemoglobin (HbA1c), and 2-h postprandial glucose. In previous studies, the positive effect of NS on the reduction of FBS and HbA1c in patients with diabetes mellitus has been well established [8–10].

There are many biochemical and clinical parameters for the evaluation of renal function. The most common are glomerular filtration rate (GFR), serum creatinine, blood urea nitrogen, uric acid, and urinary analysis. The Kidney Disease: Improving Global Outcomes organization has introduced the GFR index as a valid criterion for diagnosis and classification of renal failure. The rate of blood flow through the kidneys in the unit of time is called GFR. The amount of GFR is associated with age, sex, and body surface. Accurate measurement of GFR is complicated, as the filtration process simultaneously takes over millions of glomeruli. So, to measure it, we have to use the estimation formulas [11]. It has had more than 20 formulas for its estimation, which the most used is the Cockcroft-Gault equation [12].

Uric acid is the final product of purine metabolism during DNA and RNA synthesis. Also, the metabolism of food purine is another source of blood uric acid. This indicator is used as a criterion for kidney function. According to cohort studies with a high population, high serum uric acid is a predictor of all death reasons, especially heart disease in dialysis patients [13]. In addition, another criterion for renal function is urine volume. Low urine volume in hemodialysis patients leads to the accumulation of toxic substances in the body. Also, another complication is the cause of edema. Therefore, increasing urine volume is one of the therapeutic goals in hemodialysis patients. According to the study done on patients with diabetes mellitus with renal failure, consumption of NS oil for 3 weeks could cause a significant increase in urine volume in dialysis patients [1].

Approximately 50% of hemodialysis patients have depression and, about 25% of them suffering from severe depression [14, 15]. Several studies have shown that suicide is more prevalent among dialysis patients with depression, and their life expectancy is one third to one sixth of the normal range. Depression in ESRD patients is associated with more complications and higher mortality. Also, adherence to diet and restriction of recommended fluids was lower in ESRD patients with depression [14]. Considering the impairment of renal filtration, the benefits and risks of antidepressant drugs in ESRD patients are controversial. A scientific documentary on the positive effect of antidepressant drugs (fluoxetine, sertraline, citalopram) was not found in comparison with placebo on quality of life in ESRD patients [14]. As a result, the control and treatment of depression are one of the priorities in hemodialysis patients. The hospital scale of anxiety and depression (HADS) is a self-administered questionnaire used to assess the level of anxiety and depression in patients with physical and mental health problems. The HADS scale was first introduced by Snaith to screen psychiatric disorders in public hospitals [16]. This questionnaire has 14 questions parallel to depression and anxiety in patients. So far, many studies have been conducted on the reliability and validity of this scale in different countries and diseases. Translation of the HADS questionnaire in Persian and its standardization was conducted by Montazeri et al. in 2003 [17].

Quality of life is an important criterion that shows the effectiveness of health care, health level, and a good sense of living. In addition, it predicts the occurrence of mortality and duration of hospitalization in hospitals [18]. ESRD is a chronic limiting condition with many adverse effects on different dimensions of patients’ life [19]. RRT exposes patients to a wide range of physical, psychological, economic, and social problems and affects their quality of life. As there is no definite treatment for ESRD patients, many studies have been investigated to evaluate and improve patients’ quality of life. They have introduced this index as a therapeutic target in these patients [18]. Also, regular monitoring of this index as a clinical criterion has expressed the treatment progression of patients treated with RRT [18]. The kidney disease quality of life questionnaire (KD-QOL) is a complete version of the short form 36 health survey questionnaire (SF-36) for renal patients, as well as a self-administered questionnaire. The questionnaire has eight general dimensions for physical and mental health, as well as ten specific dimensions related to kidney disease [20]. Studies have shown a high level of consistency and internal correlation for this instrument. AH Pakpour et al. in 2011 carried out the reliability and validity of this questionnaire in Iran [19].

As mentioned in previous studies, the positive effect of NS on glycemic control in patients with diabetes mellitus and improving the renal function tests has been proven. However, there is no study about hemodialysis patients. Furthermore, quality of life and depression are the most critical qualitative indexes in hemodialysis patients; in this research, we consider the effect of NS supplementation on these indexes.

The overall aim of this design is to determine the effect of NS oil supplementation on indices of renal function, glycemic indexes, oxidative stress, inflammation, quality of life, and depression in patients with diabetes mellitus undergoing hemodialysis. Moreover, its proprietary objectives include the following: (1) determine the effect of NS oil supplementation on serum FBS, HbA1c, GA, serum insulin, insulin resistance, and the B cell function in patients with diabetes mellitus undergoing hemodialysis; (2) determine the effect of NS oil supplementation on serum levels of urea, creatinine, uric acid, and adequacy of dialysis as well as urine volume in patients with diabetes mellitus undergoing hemodialysis; (3) ascertain the effect of NS oil supplementation on superoxide dismutase (SOD), malondialdehyde (MDA), total antioxidant capacity (TAC), high-sensitivity C-reactive protein (hs-CRP) in patients with diabetes mellitus undergoing hemodialysis; and (4) determine the effect of NS oil supplements on quality of life and depression in patients with diabetes mellitus undergoing hemodialysis.

## Methods

### Study setting

The present study is a randomized clinical trial controlled with placebo and two-blinded groups that conform to standard protocol items: recommendations for interventional trials statement (SPIRIT) 2013. The study will be conducted at Imam Reza Hospital of Tabriz, Iran.

### Participants

In this study, the target population is patients with diabetes mellitus undergoing hemodialysis. The inclusion criteria are:

□ An age ≥ 20 years

□ Body mass index = 18.5–30 kg/m^2^

□ Having type 2 diabetes mellitus

□ Two or three times per week hemodialysis

□ Being on hemodialysis for at least 6 months

□ Ability and willingness to cooperate in the study

The exclusion criteria are:

- Having inflammatory or infectious diseases
- Receiving steroidal or nonsteroidal anti-inflammatory drugs
- Smoking
- Using NS oil regularly
- Changes in treatment methods (dialysis)

The hemodialysis process for all patients will be through capillary dialysis filters of polysulfone and bicarbonate dialysis solution. In case of changes in hemodialysis method or type of dialysis filters, the person will be excluded from the study.

**Fig. 1:**
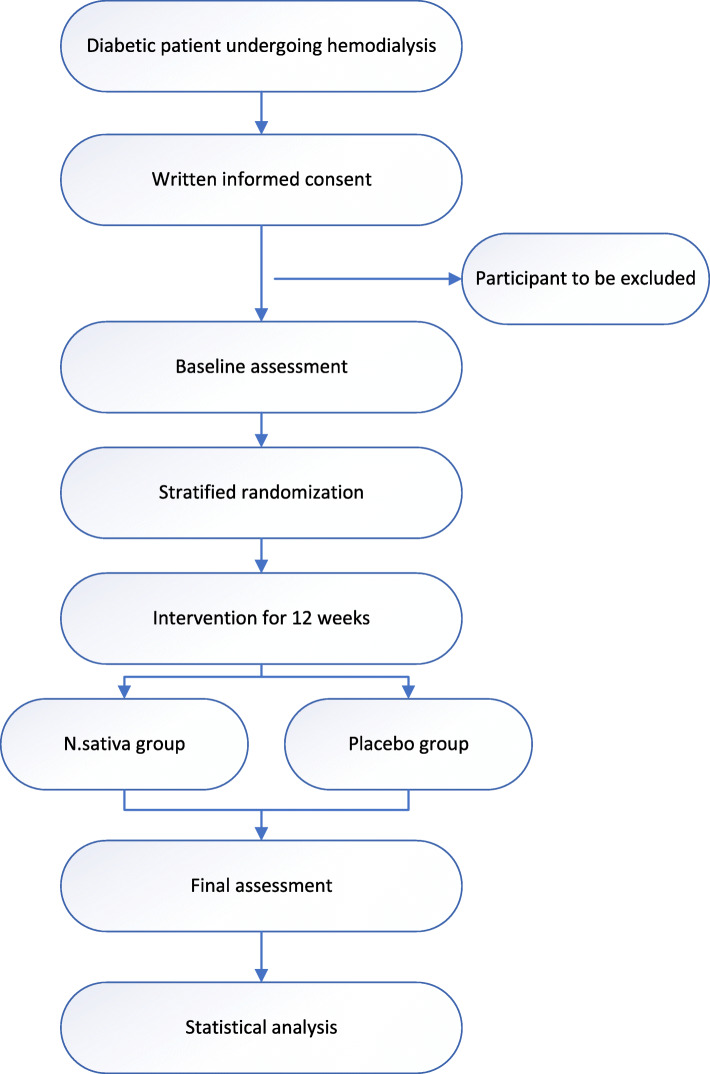
The overview of the study

The necessary information for obtaining inclusion and exclusion criteria will be extracted from hospital records, and if needed, the patient will be asked orally. The process of recruitment and randomization is illustrated in Fig. 1.

### Interventions

Following the inclusion criteria, after explaining the objectives and method of the study, patients will be consciously entered into the study. They will be randomly allocated to either the intervention or control group. Patients in the NS oil group will receive two soft gels of NS oil, whereas the control group will receive two soft gels of paraffin oil (each soft gel weights 1 g). Treatment of both groups will last for 12 weeks. The selected dose is based on Kaatabi H [8], one of the best practical and safe interventions of NS oil supplementation in patients with diabetes mellitus. According to the predominant studies, NS oil supplementation in the mentioned dose and the term, but even in more prolonged use, did not have certain complications. Just in some rare cases, mild and temporary nausea and dyspepsia and decreased appetite have been reported [9, 10, 21]. The pharmaceutical company will prepare soft gels in a completely similar way in terms of shape, color, and odor. The preparation of black seed oil will be done by the cold press method by the pharmaceutical specialist.

The supplements will be available to attendees every two weeks. All patients will be asked about their usual dietary habits, physical activities, and drug regimens and report any possible changes. In addition, every 2 weeks, all patients will be evaluated by interview. Furthermore, the researcher will give participants a call number to inform him if there are any side effects or other problems Table 1.

**Table 1:**
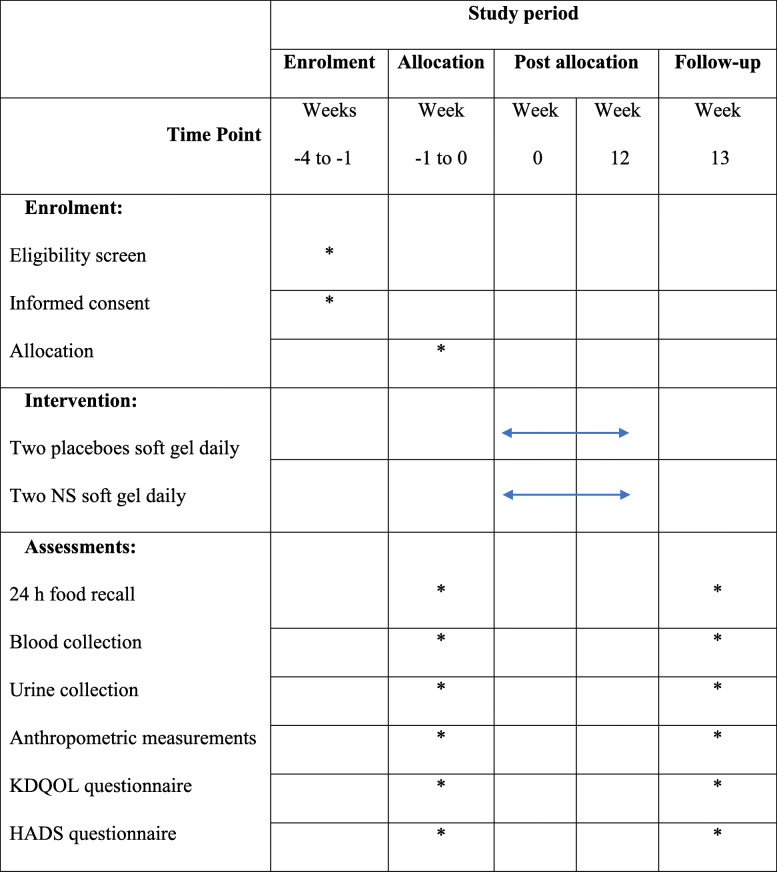
Timetable of planned activities during the study directly related to participants

Standard Protocol Items: Recommendations for Interventional Trials (SPIRIT). Schedule of enrolment, interventions, and assessments. *Abbreviations*: NS, *Nigella sativa*; KDQOL, Kidney Disease Quality of Life; HADS, Hospital Anxiety and Depression Scale

### Outcomes measures

Patients will be asked to obtain Informed consent. Next to that, everyone’s demographic and anthropometric indices checklist are filled, and a nutritionist will teach general nutritional recommendations. The dietary intake of individuals will be evaluated by the 3-day food registration questionnaire (two week days and one weekend day). Also, before the intervention, patients will be paid to complete their standard questionnaires, including KD-QOL and HADS, to assess the quality of life, depression, and anxiety. If the patient is illiterate, the information will be received by interviewing. At baseline and the end of the 12th week, 10 mL of blood will be obtained from each patient after 12 h of fasting. Blood serum will be stored at refrigerator temperature (− 70 °C).

The primary clinical outcome measures for the trial are renal function tests as creatinine, urea, uric acid, urine volume, indices related to blood glucose including FBS, HbA1c, GA, serum insulin, insulin resistance and β cell function, indices related to oxidative stress, and inflammation including SOD, MDA, TAC, and hs-CRP. Secondary outcomes include KDQOL and HADS questionnaires and the amount of energy and macronutrients intake.

The process for assessing the activity of hs-CRP, MDA, SOD, and TAC will be done by Navand assay kit (Navandsalamat, Iran) according to the company package insert instruction. The serum insulin level, HbA1c, and GA will be measured by the enzyme-linked immunosorbent assay (ELISA) method. Biochemistry Solutions will also measure creatinine, urea, uric acid, and FBS.

To determine insulin resistance and β cell function, HOMA-IR and HOMA-B formula will be used, respectively [22].

HOMA-IR=$\frac{\  glucose\left(\frac{mg}{dl}\right)\times insulin}{405}$ HOMA-B =$\frac{fasting\ insulin\times 20}{fasting\ glucose-3.5}$

The reliability of laboratory methods by sending the first ten samples of experiments with two different names to the laboratory expert and investigating the level of agreement between them will be determined. Also, body composition will be evaluated by the bioelectrical impedance analysis (BIA) method.

### Sample size

The sample size for this trial was calculated based on the primary outcome using the G-power software [23] (procedure of two independent groups) or formula $n=\frac{\left({\sigma}_1^2+{\sigma}_2^2\right){\left({Z}_{\frac{\alpha }{2}}+{Z}_{\beta}\right)}^2}{{\left({\mu}_1-{\mu}_2\right)}^2}$. According to the previous results of NS on renal failure [1], the percentage reduction in creatinine (the primary outcome) is 32.43% in the NS group and 9.84% in the placebo group. By considering an alpha error of 0.05, and the power of 90%, the minimum sample size required for each group is 20 participants, in which with predicting a 15% loss, it will be 23 patients in each group. In total, 46 participants will be recruited in this trial.

### Stratified randomization

The patients will be allocated to either a NS oil or control group by block randomization after stratification based on the frequency of hemodialysis per week (2 or 3 times per week) and the amount of blood sugar (FBS < 120 mg/dL, FBS = 120–200 mg/dL and FBS > 200 mg/dL). This process will be carried out by a statistics specialist using RAS (random allocation software) in block sizes of 4.

### Blinding

The study epidemiologist is responsible for randomization, and the study pharmacist is for blinding the supplements. The supplements, separated by letters A or B, are identical in shape, color, and odor and are placed in similar cans. So, participants, researchers, and data analysts will be blinded after determining interventions.

The blinding code will not be specified until the end of the study except in a medical emergency or a potential study-related adverse event determined by the principal investigator.

### Data management

Potential participants will be informed that the personal information collected during the study is confidential and stored safely at the University of Tabriz. Data management will be following the Data Protection Act 2018.

All questionnaires, records, and participants’ data are coded and stored correctly in locked cabinets. Only study researchers will have access to files linking a person’s study number to their name. The questionnaire, laboratory data, and medical records will be entered into the Excel database. During the study, only the principal researcher will have access to the prepared Excel files. Finally, a clean data set without identification will be generated and delivered to the biostatistician for statistical analysis. All essential documents, including source documents, will be retained at least 5 years after completing the study.

The data monitoring team will assess data quality control at the beginning and end of the study. This team monitors the exact implementation of the study based on the approved protocol, and for this purpose, they compare the data in the database with the source documents to evaluate its accuracy and completeness.

### Statistical analysis

The collected data will be analyzed by the statistical package for the social sciences (SPSS) version 23 software. Descriptive statistics, including frequency, percentage, and central indices and dispersion, will be done. The Wilcoxon signed-rank test will determine the normal distribution of the data. If data distribution is normal, to compare the serum markers and physical components analysis among the study groups in the pre-intervention stage, independent sample *t*-test, and after the intervention, analysis of covariance (ANOVA) will be used by modulating the baseline values and probable variables. A paired-samples *t*-test will be used to compare data in each study group if the data distribution is normal, compared to the serum markers and physical components analysis before the study, Mann-Whitney test, and the study groups. In that case, Wilcoxon signed-rank test will be used (for both before and after the intervention). In all tests, the *p*-value < 0.05 will be considered significant. Missing data will be removed from the final analysis. The number of dialysis sessions per week and the participants’ blood sugar levels will be matched at the beginning of the study. Other confounding factors such as energy intake, BMI, gender, and age will be adjusted if needed.

### Ethics considerations

The expert nutritionist will explain the purpose of the study, benefits, or side effects to all patients before obtaining written informed consent. The volunteers will fill an informed consent form and enrolled in the study. They can withdraw their consent at any time study.

According to previous studies, this supplement’s use is safe in the dose, as mentioned earlier, and has no side effects. In case of any acute clinical symptoms due to supplementation, the subjects will be excluded, and the researcher will pay the costs of any possible complication. Any adverse events and other unintended effects of trial interventions will be collected and mentioned in the article.

The personal information of enrolled participants will be collected, shared, and confidentiality maintained before, during, and after the study by encoding systems. The results of the experiments will be available for participants at the end. The participants will be assured that the project will not be charged for treatment interventions. If NS supplementation is effective, the control group will also take this supplement.

The benefits of participating in this study for the control group are free:

- Nutritional counseling and monthly monitoring
- Glycemic tests at the beginning and end of the study
- Kidney function tests at the beginning and end of the study
- Inflammation and oxidative stress status tests at the beginning and end of the study
- Body composition and anthropometric tests at the beginning and end of the study

All results will be delivered to the participants at the end of the study.

## Discussion

Diabetes and chronic kidney disease are the most common diseases of the present century that have an increasing prevalence. Diabetes is the leading cause of diabetic kidney disease (DKD), leading to ESRD and the onset of dialysis or kidney transplantation [1]. The prevalence of RRT is increasing in many countries, and the number of dialysis patients is estimated to reach at least 5.4 million by 2030 [4]. Since there is no definitive and conventional treatment for ESRD patients, treatment in these patients aims to prevent existing complications and improve their quality of life [24].

*Nigella sativa* is a healing plant with positive effects on renal and blood glucose indices in previous experimental and human studies [1, 10]. Also, positive evidence has been reported about improving memory and quality of life and reducing depression, which are common problems in hemodialysis patients. Despite this, no trial has been done on hemodialysis patients.

Considering the relatively low cost and very low complications of this intervention, if significant results are obtained in this study, it can be used as an adjective therapy in these patients. We designed this study to investigate the effect of *Nigella sativa* oil supplementation on the mentioned indices in hemodialysis patients.

## Trial status

The first-stage sampling of patients is almost finished. The protocol has been submitted before the last patient. The first participant’s enrollment to the trial was on 1 June 2020, and it is estimated that primary sampling is completed by 20 August 2020.

Trial registration: Iranian Registry of Clinical Trials: IRCT20200411047027N1. Registered on 31 May 2020, https://fa.irct.ir/user/trial/48113/view

## Supplementary Information

**Additional file 1.** SPIRIT 2013 checklist (revised): recommended items to address in a clinical trial protocol and related documents.

**Additional file 2.** Consent form used in this trial.

## Data Availability

The datasets generated during this study will be available via the corresponding author on a reasonable request.

## Abbreviations

ANOVA: Analysis of variance
BMI: Body mass index
CKD: Chronic kidney disease
CVD: Cardiovascular disease
ESRD: End-stage of renal disease
GA: Glycosylated albumin
HADS: Hospital Anxiety and Depression Scale
HbA1c: Glycosylated hemoglobin
HOMA-B: Homeostatic model assessment of beta-cell function
HOMA-IR: Homeostatic model assessment of insulin resistance
hs-CRP: High-sensitivity C-reactive protein
I.R: Insulin resistance
KD-QOL: Kidney disease quality of life
N.S: *Nigella sativa*
RRT: Renal replacement therapy
S.G: Specific gravity
SOD: Superoxide dismutase
MDA: Malondialdehyde
T2DM: Type 2 diabetes mellitus
TAS: Total antioxidant capacity
