# Supplementary material for: Effect of Nigella sativa supplementation on kidney function, glycemic control, oxidative stress, inflammation, quality of life, and depression in diabetic hemodialysis patients: study protocol for a double-blind, randomized controlled trial
Source: Trials. 2022 Feb 4;23:111. doi: 10.1186/s13063-021-05917-y (PMC8815251; doi:10.1186/s13063-021-05917-y)
Supplement: Supplementary file 2 — Additional file 2. Consent form used in this trial. [file 13063_2021_5917_MOESM2_ESM.pdf]

رضایت نامه ورود به طرح تحقیقاتی

عنوان/موضوع تحقیق: تأثیر مکمل‌یاری روغن سیاه دانه بر شاخص‌های عملکرد کلیه، شاخص‌های قند خون، استرس اکسیداتیو، وضعیت التهاب، کیفیت زندگی و افسردگی در بیماران دیابتی تحت درمان با همودیالیز
نوع تحقیق: کارآزمایی بالینی تصادفی دو سو کور

آقا/خانم محترم
از شما دعوت می شود در یک مطالعه تحقیقاتی که توسط دانشگاه علوم پزشکی تبریز تحت نظارت کمیته منطقه ای اخلاق دانشگاه انجام یافته و توسط معاونت پژوهشی داتشگاه تامین مالی می شود شرکت کنید. پیش از آنکه تصمیم به شرکت یا عدم شرکت بنمائید، بطور خلاصه تحقیق برای شما توضیح داده میشود که چرا این تحقیق صورت می گیرد و این تحقیق مستلزم چه چیزی است. لطفا در خواندن اطلاعات زیر در مورد تحقیق عجله نکنید و آنها را بدقت بخوانید. هر کجا نیاز به توضیح داشتید سوال فرمایید و درباره تصمیم در مورد شرکت یا عدم شرکت خودتان عجله نکنید.
هدف این مطالعه چیست؟ و چگونه انجام خواهد شد؟
هدف مطالعه حاضر بررسی تعیین اثر مکمل روغن سیاه دانه بر شاخص‌های عملکرد کلیه، شاخص‌های قند خون،استرس اکسیداتیو، التهاب ، کیفیت زندگی و افسردگی در بیماران دیابتی تحت درمان با همودیالیز میباشد. برای افراد شرکت کننده پس از دارا بودن معیارهای ورود، پرسشنامه مشخصات فردی تکمیل خواهد شد. در ابتدا و انتهای مطالعه ۱۰ میلی لیتر خون درحالت ناشتا از بیماران گرفته و پس از سانتریفیوژ در دمای$^{\circ}$۷۰- نگهداری خواهند شد. پرسشنامه ثبت غذایی ۳ روزه (دو روز کاری و یک روز تعطیل)، فعالیت فیزیکی و میزان افسردگی و کیفیت زندگی برای شرکت کنندگان در ابتدا و انتهای مطالعه تکمیل خواهد شد. قبل و بعد از دوره مکمل یاری آزمایشات مربوط به کنترل قند خون، عملکرد کلیه، سیستم آنتی اکسیدانی و التهابی انجام خواهد شد. اندازه گیری های تن سنجی شامل وزن، قد برای افراد در ابتدا و انتهای مطالعه اندازه گیری و ثبت میشوند.
چرا من انتخاب شده ام؟
بدلیل مشخصات فردی و دارا بودن معیارهای ورود به صورت داوطلبانه میتوانید وارد مطالعه شوید.
منافع این تحقیق چیست؟
با توجه به عوارض زیادی که بیماران تحت درمان با همودیالیز مستعد آن هستند، مکمل‌یاری با سیاه دانه میتواند به عنوان یک درمان کمکی خوب در بهبود وضعیت بیمار موثر واقع شود.
آیا خطر و یا عوارض احتمالی نیز در کار خواهد بود؟ خیر
آیا شرکت من در این مطالعه محرمانه خواهد ماند؟
شرکت شما در این مطالعه و اطلاعات/ داده هایی که شما در اختیار من می گذارید، کاملا محرمانه باقی خواهد ماند. یک شماره یا کد شناسایی در طول مطالعه برای هر یک از شرکت کنندگان اختصاص یافته و تمام داده ها ناشناخته باقی خواهند ماند. در مورد داده ها مطابق با قوانین مراقیت از داده ها در ایران که محرمانه بودن آنها را تضمین می کند عمل خواهد شد.
اگر بخواهم شرکت کنم چه کاری باید انجام دهم؟
اگر شما برای شرکت در این مطالعه موافقت کنید، لازم است فرم رضایت آگاهانه را تکمیل نموده و به محقق برگردانید . لطفا این برگ حاوی اطلاعات را برای خود نگه دارید. اگر تصمیم به شرکت در این تحقیق گرفتید، هر زمانی شما مجاز هستید از این تحقیق کناره‌گیری کنید بدون اینکه دلیلی برای ما اقامه نمایید.

در این بخش به طورخلاصه توضیحی در مورد نحوه مشارکت و نقش مشارکت‌کننده در تحقیق داده شود:

در ابتدای مطالعه چند پرسش‌نامه برای شما تکمیل خواهد شد. وزن و قد شما اندازه گیری شده و ده سی‌سی خون ناشتا از شما اخذ می‌گردد. نتیجه آزمایش همراه با مکمل‌ها به شما تحویل داده می‌شود. هر دو هفته یکبار به متخصص تغذیه مراجعه می‌نمایید تا مکمل‌های دو هفته بعدی را در اختیار شما بگذارد. ۱۲ هفته پس از شروع مطالعه، چند پرسش‌نامه برای شما تکمیل می‌شود و دوباره ده سی سی خون از شما اخذ می‌شود. نتیجه آزمایش به شما ارائه داده می‌شود. ارائه تمام این رژیم‌های غذایی و مکمل‌ها و نیز آزمایش خون برای شما به صورت رایگان انجام خواهند شد.

اگر شما سوالی دارید و یا اینکه مایل به اطلاعات بیشتری هستید، لطفا با علی طریقت اسفنجانی شماره تلفن: ۰۹۱۴۳۰۰۵۸۹۵ یا E-mail:tarighat٤٥@gmail.com تماس بگیرید.

با تشکر از وقت شما برای قبول زحمت خواندن این برگ.

رضایت آگاهانه

کد / شماره مطالعاتی:

عنوان تحقیق: تعیین اثر مکمل روغن سیاه دانه بر شاخص‌های عملکرد کلیه، شاخص‌های قند خون،استرس اکسیداتیو، التهاب ، کیفیت زندگی و افسردگی در بیماران دیابتی تحت درمان با همودیالیز

لطفاً علامت‌گذاری کنید:

۱- من تأیید می‌کنم که برگ اطلاعات مشارکت‌کننده به تاریخ را برای انجام تحقیق فوق خوانده و فهمیده‌ام و این فرصت برای من داده شده که سؤالات مورد نظرم را بپرسم. ☐

۲- من می‌دانم که شرکت من در این تحقیق داوطلبانه است. من همچنین می‌دانم که من هر زمانی که بخواهم می‌توانم از تحقیق کنار بکشم بدون اینکه ملزم به ارائه دلیل باشم. ☐

۳- من موافقت می‌کنم که در مطالعه/ تحقیق فوق شرکت نمایم. ☐

نام مشارکت‌کننده تاریخ: امضاء

نام محقق تاریخ: امضاء
